# A theoretical analysis of the reward rate optimality of collapsing decision criteria

**DOI:** 10.3758/s13414-019-01806-4

**Published:** 2019-07-29

**Authors:** Udo Boehm, Leendert van Maanen, Nathan J. Evans, Scott D. Brown, Eric-Jan Wagenmakers

**Affiliations:** 1 Department of Experimental Psychology, University of Groningen, Grote Kruisstraat 2/1, 9712TS, Groningen, The Netherlands; 2 Department of Psychology, University of Amsterdam, 1018 XA, Amsterdam, The Netherlands; 3 School of Psychology, University of Newcastle, Callaghan, NSW 2308 Australia

**Keywords:** Collapsing bounds, Diffusion model, Reward rate maximization

## Abstract

A standard assumption of most sequential sampling models is that decision-makers rely on a decision criterion that remains constant throughout the decision process. However, several authors have recently suggested that, in order to maximize reward rates in dynamic environments, decision-makers need to rely on a decision criterion that changes over the course of the decision process. We used dynamic programming and simulation methods to quantify the reward rates obtained by constant and dynamic decision criteria in different environments. We further investigated what influence a decision-maker’s uncertainty about the stochastic structure of the environment has on reward rates. Our results show that in most dynamic environments, both types of decision criteria yield similar reward rates, across different levels of uncertainty. This suggests that a static decision criterion might provide a robust default setting.

## Introduction

Considerations of what constitutes optimal behavior have long played a prominent role in research on human decision-making (e.g., Kahneman and Tversky (1979) and Savage (1954)). Arguments based on economic optimality have traditionally focused on economic decisions, where decision-makers choose among different options based on their associated rewards (Summerfield and Tsetsos, 2012). However, in recent years economic arguments have also gained attention in the area of perceptual decision-making, where decision-makers have to choose among different interpretations of a noisy stream of sensory information. The process by which an interpretation is chosen is often characterized as a sequential sampling process; decision-makers first set a static decision criterion, a fixed amount of information they require to commit to a decision, and subsequently accumulate sensory information until that criterion is reached (Ratcliff, 1978; Ratcliff & Smith, 2004; Edwards, 1965; Heath, 1981; Stone, 1960).

Recently, a number of authors have argued that perceptual decision-making is governed by reward rate (RR) optimality, which means that decision-makers aim to maximize their expected rewards per unit time (Cisek et al., 2009; Drugowitsch et al., 2012; Shadlen & Kiani, 2013; Thura et al., 2012). As detailed below, RR optimality implies that a static decision criterion will yield maximal rewards if certain aspects of the decision environment, such as task difficulty and rewards, remain constant over time. However, if these aspects of the decision environment vary dynamically, decision-makers need to dynamically adjust their decision criterion to obtain maximal rewards. Proceeding from the assumption that decision environments are typically dynamic, Cisek et al., (2009), Shadlen and Kiani (2013), Thura et al., (2012) have argued that a dynamic decision criterion that decreases over time should replace the standard assumption of a static criterion. This economic optimality argument has received much attention in the literature and has been incorporated into formal models of perceptual decision-making (Huang and Rao, 2013; Rao, 2010; Standage et al., 2011). However, reviews of the existing literature and published data suggest that the empirical support for an axiomatic decreasing decision criterion is considerably weaker than claimed by its proponents (Boehm et al., 2016; Evans et al., 2017a; Hawkins et al., 2015; Voskuilen et al., 2016). Moreover, studies that provide support for a decreasing decision criterion often make additional ad hoc assumptions that complicate the interpretation of theoretical and empirical results (Boehm et al., 2016).

Evans et al., (2017a), for instance, provide an extensive discussion of Cisek et al.’s (2009) urgency gating model (UGM), which implements a dynamic decision criterion, in comparison to Ratcliff’s (1978) diffusion model (DM), which implements a static decision criterion. As Evans et al., point out, both models make markedly different behavioral predictions. However, the relationship between these behavioral predictions and the type of decision criterion each model uses is not clear. Although both models share the core assumption of a Gaussian evidence accumulation process, they differ in several other assumptions that are not related to the decision criterion but may critically influence the behavioral predictions. Cisek et al., (2009) and Thura et al., (2012), on the other hand, emphasize the role of the dynamic decision criterion for differences in predicted RR between the UGM and the DM. Evans et al., (2017a) further find that studies in support of the UGM typically use heuristic reasoning, and the conclusions from this reasoning often do not match the actual predictions of the model. Boehm et al., (2016) note similar shortcomings in studies that compare other implementations of a dynamic decision criterion to a static decision criterion. In the present work, we will implement dynamic and static decision criteria in a common framework and we will carry out a systematic theoretical analysis for a typical experimental design to evaluate claims that a decreasing dynamic criterion is generally RR optimal.

The criterion that is typically invoked to decide whether a static decision criterion or a dynamic decision criterion is RR optimal is the dynamics of the decision environment. In a static task environment in which all trials are equally difficult (i.e., all stimuli are equally noisy) and the reward for a correct decision remains constant over time, RR optimality can be achieved using a static decision criterion. Specifically, because task difficulty is constant across trials, the expected decision time under a static decision criterion is the same for all trials and can be minimized for a given accuracy level by appropriately setting the static decision criterion, thus maximizing RR (Bogacz et al., 2006; Moran, 2015; Wald and Wolfowitz, 1948; Wald, 1945). However, in a dynamic task environment where some trials are very difficult and other trials are relatively easy but the reward for a correct decision remains constant, a static decision criterion is no longer optimal. Because task difficulty varies across trials, the expected decision time under a static decision criterion is shorter for easy trials, and longer for very difficult trials. By decreasing the decision criterion as time passes, decision-makers can reduce the time they spend on hard trials and instead attempt a new trial that is likely to be easier, and thus more likely to yield a reward in a short amount of time (Shadlen & Kiani, 2013; Cisek et al., 2009; Thura et al., 2012). Therefore, in situations with constant rewards and mixed trial difficulties, a dynamic decision criterion should be RR optimal whereas in situations with constant rewards and fixed trial difficulties a static decision criterion should be RR optimal.

A further factor of influence on the optimal decision criterion are sampling costs (Drugowitsch et al., 2012; Busemeyer & Rapoport, 1988; Rapoport & Burkheimer, 1971). In the decision environments considered above, decision-makers receive a fixed reward for a correct decision and the dynamics of the environment are determined by whether task difficulty remains constant over time. A second way in which a decision environment can be dynamic is if the decision-maker’s total reward is time-dependent, which can be implemented through the addition of sampling costs to a fixed reward for correct decisions. Sampling costs are costs a decision-maker incurs by delaying the final decision by a time step to collect additional sensory information. Depending on the specific cost function, sampling costs can render either increasing or decreasing dynamic decision criteria RR optimal.

Despite its intuitive appeal, the categorization of the decision environment in terms of the dynamics of task difficulty and sampling costs provides an incomplete account of human decision behavior. Empirical studies that create a dynamic decision environment regularly fail to elicit a dynamic decision criterion. For example, in lexical decision tasks participants are typically presented a mixture of high- and low-frequency words, where high-frequency words can be considered easy stimuli and low-frequency words can be considered hard stimuli. Although data from lexical decision tasks have for many years been analyzed using the diffusion model (Ratcliff (1978)), which relies on a static decision criterion, no studies have reported any systematic discrepancies between model and data (e.g., Yap et al., (2015), Ratcliff and Smith (2004), and Wagenmakers et al., (2008)). Similarly, in a recent study using numerosity judgment and motion discrimination tasks, a mixture of difficulties failed to reliably elicit a dynamic decision criterion (Voskuilen et al., 2016).

A possible reason for this failure of the categorization in terms of dynamics is that using a static decision criterion in a dynamic decision environment might only yield a negligibly lower RR than the optimal dynamic decision criterion (Ditterich, 2006), and therefore provide insufficient motivation for decision-makers to adapt their decision criterion. Moreover, decision-makers usually do not have full knowledge of the stochastic properties of the decision environment but need to build an internal representation based on repeated interactions with the decision environment. The uncertainty inherent in such an experience-based representation might further diminish the RR gained by adopting an optimal decision criterion compared to a suboptimal static decision criterion. In the present study, we will investigate the influence of the dynamics of the decision environment and of uncertainty about the stochastic structure of the environment on RR optimality. To this end, we will show how, in a typical experimental paradigm, the expected RR under the optimal dynamic decision criterion and under a static decision criterion behave as a function of the stochastic structure of the decision environment and the decision-maker’s uncertainty about this structure.

## Decision environment and sequential sampling model

For our theoretical analysis, we will consider a type of decision environment that is typically created in expanded judgment tasks (Irwin et al., 1956). In these tasks, participants are presented stimuli that consist of a series of discrete events of fixed duration. Each event is sampled from a set of possible events according to a probability distribution and participants are asked to make inferences about the probability distribution; in most cases they are asked to decide which of the events has the highest probability of occurring. For instance, participants might be shown two circles that flash at different rates and be asked to decide which circle flashes at a higher rate (e.g., Sanders and Ter Linden (1967) and Wallsten (1968)). One major advantage of expanded judgment tasks over other types of decision tasks is that they allow researchers to directly track decision-makers’ current state of evidence. Given the events presented to the decision-maker up to a specific point in time, researchers can easily compute the posterior probability of one event type having a higher probability of occurring than the other event types. The posterior probability at the time of decision commitment then gives an approximation of the decision-maker’s decision criterion.

### Stochastic structure of the decision environment

The type of experimental task we will analyze here is a two-alternative forced choice (2AFC) task in which participants might, for instance, be presented two visual stimuli, one on the left side of the screen and one on the right. Each stimulus consists of a sequence of sensory events that are presented in fixed intervals. Each sensory event consists of either the presence of sensory information, a positive event, or the absence of sensory information, a negative event. If stimuli consist of a series of light flashes, for instance, the occurrence of a flash is a positive event whereas the absence of a flash is a negative event. The events that constitute a stimulus are sampled independently from the positive or negative category according to a probability distribution that is specific to each stimulus. In particular, for one of the two stimuli, the target, the rate *𝜃*_*T*_ of a positive event is higher than for the other stimulus, the distractor, for which positive events are sampled with rate *𝜃*_*D*_. The sampling of the events for each stimulus thus constitutes a series of independent Bernoulli trials and the decision-maker’s task is to decide for which of the two stimuli the rate parameter is higher.

Because the events for both stimuli are sampled independently, there are four types of observations the decision-maker might make. These observations constitute a random variable *X* with values *x* ∈{(1,0),(0,1),(1,1),(0,0)}. First, a positive event might be sampled for the target stimulus but not for the distractor (e.g., the target flashes but not the distractor), in which case the decision-maker observes evidence for the target having the higher rate parameter and *X* = (1,0). The probability of this occurring is *p* = *𝜃*_*T*_(1 − *𝜃*_*D*_). Second, a positive event might be sampled for the distractor but not for the target (e.g., the distractor flashes but not the target), in which case the decision-maker observes evidence for the distractor having the higher rate parameter and *X* = (0,1). The probability of this occurring is *q* = (1 − *𝜃*_*T*_)*𝜃*_*D*_. Note that our assumption that *𝜃*_*T*_ > *𝜃*_*D*_ implies *p* > *q*. Third, a positive event might be sampled for both stimuli (e.g., both stimuli flash), in which case *X* = (1,1) and the probability of this event is *𝜃*_*T*_*𝜃*_*D*_. Finally, a negative event might be sampled for both stimuli (e.g., no stimulus flash), in which case *X* = (0,0) and the probability of this event is (1 − *𝜃*_*T*_)(1 − *𝜃*_*D*_). Note that although the last two types of observations do not convey information about how the rate parameters differ between the two stimuli, they do provide information about the rate at which events occur in general. This type of information is essential when decision-makers have incomplete knowledge of the structure of the task environment and need to infer the rate parameters for the two stimuli from their interactions with the task environment.

### Sequential sampling model

The standard way of modeling the type of 2AFC task just described is in terms of a sequential sampling problem in which the decision-maker entertains two competing hypotheses (Rapoport and Burkheimer, 1971; Ratcliff, 1978). The first hypothesis, $\mathcal {H}_{l}$, states that the left stimulus is the target. The second hypothesis, $\mathcal {H}_{r}$, states that the right stimulus is the target. Each hypothesis $\mathcal {H}_{i}, i \in \{l,r\}$, implies a likelihood function *λ*_*i*_(*x*) for the observations of *X*. The likelihood function under $\mathcal {H}_{l}$ is:

1 $$  \lambda_{l}(x)= \left\{\begin{array}{ll} \theta_{T}(1-\theta_{D}) & \text{if } x=(1,0)\\ \theta_{D}(1-\theta_{T}) & \text{if } x=(0,1)\\ \theta_{T}\theta_{D} & \text{if } x=(1,1)\\ (1-\theta_{T})(1-\theta_{D}) & \text{if } x=(0,0) \end{array}\right.. $$

Due to the symmetry of the hypotheses, the likelihood function under $\mathcal {H}_{r}$ is the same as the likelihood function under $\mathcal {H}_{l}$ with the roles of *𝜃*_*T*_ and *𝜃*_*D*_ reversed.

Before observing any events, the decision-maker might hold a prior belief *π*(0) that $\mathcal {H}_{l}$ is true. We will assume here that the decision-maker is unbiased, that is, *π*(0) = 0.5. The decision-maker subsequently observes a series of discrete events *x*_*t*_ at time steps *t* ∈{1,…,*N*} and updates the prior belief after each observation according to Bayes’ rule:

2 $$ \pi(t)=\frac{\pi(t-1)\lambda_{l}(x_{t})}{\pi(t-1)\lambda_{l}(x_{t})+(1-\pi(t-1))\lambda_{r}(x_{t})}. $$

After each observation the decision-maker faces a choice between three options (cf. Wald’s 1945) sequential probability ratio test). First, decide that $\mathcal {H}_{l}$ is true, second, decide that $\mathcal {H}_{r}$ is true, or, third, postpone the final decision and wait for an additional observation. This choice is governed by the decision-maker’s decision criterion. If the posterior belief *π*(*t*) after the *t* th observation that $\mathcal {H}_{l}$ is true exceeds a certain upper criterion value, *δ*_*l*_(*t*), the final decision is made immediately that $\mathcal {H}_{l}$ is true. If *π*(*t*) falls below a certain lower criterion value, *δ*_*r*_(*t*), the final decision is made immediately that $\mathcal {H}_{r}$ is true. Because we assume that rewards depend only on the accuracy of the decision but not the specific stimulus chosen (see next section), the upper and lower decision criterion are symmetric around 0.5, that is, *δ*_*r*_(*t*) = 1 − *δ*_*l*_(*t*), and it suffices to consider only one decision criterion. If the posterior probability after the *t* th observation exceeds neither decision criterion, the final decision is postponed by at least one time step.

## Reward rate optimal decision criterion

According to the RR hypothesis, decision-makers should choose a decision criterion that maximizes their expected RR. The specific shape of the RR optimal decision criterion depends on the structure of the task environment and the decision-maker’s knowledge of this structure. Here we will consider three factors that influence the shape of the RR optimal decision criterion. We already mentioned the role the dynamics of the decision environment play in determining the shape of the optimal decision criterion. In a task environment with constant total rewards and constant difficulties across trials, a decision criterion that remains constant throughout the decision process (i.e., a static decision criterion) will yield the maximal RR (Wald, 1945). On the other hand, if the decision environment is dynamic, either due to a variable task difficulty or due to a time-dependent total reward, a criterion that changes over the course of the decision process (i.e., a dynamic decision criterion) is optimal (Frazier & Yu, 2008; Rapoport & Burkheimer, 1971). Here we will focus on the effect variable sampling costs have on the shape of the optimal decision criterion as this allows for a relatively straightforward mathematical analysis. A discussion of the effect of variability in task difficulty can be found in Malhotra et al., (2018).

The second factor we will consider is the overall difficulty of the experimental task. Although we assume that task difficulty is constant, a higher overall task difficulty means that correct decisions require more observations. This introduces a trade-off between the time spent on a decision and the probability of earning a reward, which should be reflected in the shape of the RR optimal decision criterion.

The third factor we will consider is uncertainty about the structure of the decision environment. Uncertainty may concern several aspects of the experimental task, such as the rate parameters of the target and distractor stimulus, response deadlines, or the sampling costs the decision-maker has accrued at a given point in time. However, many sources of uncertainty can be controlled experimentally. Uncertainty about response deadlines and sampling costs, for instance, can be eliminated by explicitly displaying the remaining time and the accrued sampling costs. We will therefore focus on the effect uncertainty about the rate parameters of the target and distractor stimulus has on the shape of the RR optimal decision criterion.

### Formal definition of reward rate

Reward rate can be generally defined as (Drugowitsch et al., 2012):

3 $$ RR = \frac{\langle R \rangle - \langle C(T_{d}) \rangle}{\langle T_{t} \rangle + \langle t_{i} \rangle + \langle t_{p} \rangle},  $$

where 〈⋅〉 indicates the average over choices, decision times, and values of *t*_*i*_ and *t*_*p*_. 〈*R*〉 is the average reward for the final decision. 〈*C*(*t*_*d*_)〉 denotes the average total sampling costs at decision time *T*_*d*_. These are the costs a decision-maker incurs by postponing the final decision by at least one time step to observe an additional sensory event. The sampling costs at each point during the decision process are given by the cost function *c*(*t*) and a decision-maker who gives a final decision after *T*_*d*_ time steps will have to pay total sampling costs $C(T_{d}) = {\sum }_{t=1}^{T_{d}} c(t)$.

The quantities in the denominator in Eq. 3 represent the effect of temporal discounting; rewards and sampling costs affect RR less strongly as they are accumulated over a longer period of time. 〈*T*_*t*_〉 is the expected total duration of each trial, 〈*t*_*i*_〉 is the average inter-trial interval and 〈*t*_*p*_〉 is the average punishment delay imposed for incorrect responses. Note that this formulation of RR differentiates between the decision time *T*_*d*_ and the total trial duration *T*_*t*_; although the decision-maker’s accumulated sampling costs depend on *T*_*d*_, the trial might continue without further sampling costs for an additional period of time *T*_*t*_ − *T*_*d*_ after the decision-maker has indicated a final decision.

In this general form, RR depends on a number of factors that complicate the derivation of the optimal decision criterion and are not an essential part of expanded judgment tasks. We will therefore introduce some simplifying assumptions that make the formulation more amenable to our theoretical analysis. First, we will assume that all trials have the same length *T*_*t*_, independent of the decision-maker’s decision time *T*_*d*_, and that the inter-trial interval *t*_*i*_ is fixed. Second, we will assume that there is no punishment delay *t*_*p*_ associated with incorrect responses. With these simplifications in place, the denominator in Eq. 3 becomes a constant and decision-makers can maximize RR by maximizing the expected net rewards in the numerator.

Given the sequential sampling model and the structure of the experimental task with parameters *𝜃*_*T*_ and *𝜃*_*D*_ and a cost function *c*(*t*), the optimal decision criterion can now be derived using dynamic programming techniques (Bellman, 2003; Rapoport and Burkheimer, 1971). In what follows, we will first show how the RR optimal decision criterion is affected by sampling costs and task difficulty under the sequential sampling model outlined above, where it is assumed that the decision-maker has complete knowledge of the stochastic structure of the decision environment. We will subsequently modify our sequential sampling model to include uncertainty about the stochastic structure and show how this uncertainty affects the RR optimal decision criterion. Moreover, we will compare the RR optimal dynamic decision criterion to the best static decision criterion, which yields the highest RR among all possible static decision criteria.

### Influence of sampling costs

We consider two different reward schemes and the optimal decision criteria they imply. Both reward schemes have in common that the decision-maker receives a constant reward of 1000 points for correct decisions and a constant penalty of -500 points for incorrect decisions. In addition, the decision-maker incurs sampling costs every time the final decision is postponed by one time step. Under the first reward scheme, additional observations become more expensive as time passes, that is, sampling costs increase. Under the second reward scheme, additional observations become cheaper as time passes, that is, sampling costs decrease. We implement these two reward schemes using a logistic cost function that we parameterize so that, over the course of 30 observations, the total sampling costs accrue to 500 points. Together with the fixed rewards and penalties for correct and incorrect decisions, this choice of the cost function implies that, after 30 observations, the expected reward for guessing is 0 points. We will furthermore assume that the decision-maker has to commit to a final decision after 30 time steps and not deciding will result in a penalty of -1000 points (i.e., the total sampling costs for 30 time steps plus the penalty for an incorrect response). For the increasing costs case the cost function is:

4 $$ c(t)=\frac{74.92217}{1+e^{3-(t/10)}} $$

and the function for the decreasing costs case is obtained by replacing *t* by 31 − *t*, which means that the function is traversed in the opposite direction. Our choice of the logistic cost function was based on a specific experimental setup in which the accumulated sampling costs were displayed in real time. Sampling costs had to grow non-linearly for decision-makers to be able to clearly identify changes in the speed at which sampling costs grew at the start of a trial compared to the end of a trial. Nevertheless, as the argument below shows, a large class of monotonically increasing or decreasing cost functions will lead to qualitatively similar results.

**Fig. 1:**
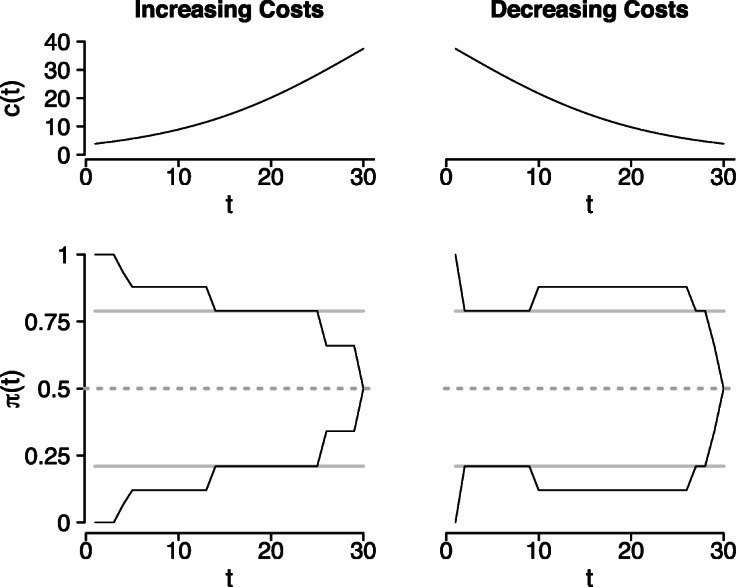
Cost functions and example static and dynamic decision criteria. The *top panels* show the functions determining the sampling costs for an additional observation at time step *t*. In the *left panel* the costs increase as time passes, in the *right panel* the costs decrease as time passes. The *bottom panels* show the optimal, dynamic decision criteria for each cost function as *solid lines*. The best constant, static decision criteria are shown as *gray lines*. The decision criteria shown are the optimal dynamic and best static criteria for *𝜃*_*T*_ = 0.38 and *𝜃*_*D*_ = 0.24

We will focus on an intuitive account of the different effects of the two cost functions on the optimal dynamic decision criterion here. A formal description of the dynamic programming techniques used to derive the optimal decision criterion is presented elsewhere (e.g., DeGroot (1969) and Rapoport and Burkheimer (1971)). Figure 1 shows the cost function (top panels) and optimal dynamic decision criteria (solid lines, bottom panels) for the increasing cost case (left) and the decreasing cost case (right) with *𝜃*_*T*_ = 0.38 and *𝜃*_*D*_ = 0.24. The jagged appearance of the decision criteria is due to the discrete time steps and evidence units in the experimental task considered here. Decision-makers update their posterior beliefs after each new observation, which are presented at fixed time intervals. Moreover, because the number of possible observations at any time is finite, the posterior belief is updated in discrete steps.

As can be seen in the bottom left panel of Fig. 1, increasing sampling costs lead to a dynamic decision criterion that collapses quickly toward 0 as time passes. This result can be intuitively understood in terms of a trade-off between the chances of making a correct decision and the mounting costs of waiting. Assuming that the left stimulus is indeed the target (i.e., $\mathcal {H}_{l}$ is true), as the decision-maker waits longer to make a final decision, the posterior probability for $\mathcal {H}_{l}$ will slowly increase. Therefore, the expected reward, which is 1000 ⋅ *π*(*t*) − 500 ⋅ (1 − *π*(*t*)), will also slowly increase. However, at the same time the total sampling costs increase at an ever higher rate, thus increasingly offsetting the small gains in expected reward as time passes. Consequently, the decision-maker stands to gain less and less from a correct decision but risks losing more and more for an incorrect decision, and should therefore become increasingly willing to risk an incorrect decision while it is still relatively cheap.

Decreasing sampling costs, on the other hand, lead to a non-monotonic dynamic decision criterion that increases as time passes but eventually collapses toward 0 at the decision deadline (see bottom right panel of Fig. 1). This result can again be understood in terms of a trade-off between the chances of making a correct decision and the costs of waiting. As the decision-maker gathers more observations from the stimuli, the posterior probability for $\mathcal {H}_{l}$ increases, and so does the expected reward. Although the total sampling costs also increase, they do so at a decreasing rate. Consequently, the increase in expected reward increasingly dominates the trade-off and the decision-maker should become increasingly willing to risk a tiny additional loss for an incorrect decision while losing relatively little of the increase in expected reward by waiting for an additional time step.

The solid gray lines in the bottom panels of Fig. 1 show the RR optimal static decision criteria for the two reward schemes. As can be seen, the best static decision criterion in the increasing costs case (left panel) intersects the optimal dynamic decision criterion after about half of the available time for the decision process and subsequently stays above the optimal criterion. This might suggest that a static decision criterion leads the decision-maker to wait too long before committing to a final decision, thus losing expected rewards due to staggering sampling costs. In the decreasing costs case (right panel), the best static decision criterion intersects the optimal criterion repeatedly and the differences between the two criteria appear to be relatively small except at the time of the decision deadline when the optimal criterion collapses to 0.5. In this case, the decision-maker will tend to commit to a final decision at similar times and evidence values under the static decision criterion as under the optimal criterion. The decision performance before the deadline might, therefore, be expected to be near-optimal even under a static decision criterion. However, for both reward schemes the best static decision criterion remains at a high value at the time of the decision deadline and will therefore incur certain loss if the posterior probability has not reached the decision criterion. The optimal dynamic decision criteria, on the other hand, collapse towards 0.5 before the decision deadline, which avoids certain loss due to the penalty for a late response. These qualitative considerations suggest that differences between static and dynamic decision criteria might only result in markedly different RRs in the case of increasing sampling costs or if task difficulty is very high.

### Influence of task difficulty

As described above, our decision environment is characterized by the two rate parameters *𝜃*_*T*_ and *𝜃*_*D*_ that determine the likelihood functions *λ*_*i*_(*x*) under the two competing hypotheses. The decision-maker uses the observed stimulus events to update the belief *π*(*t*) about which stimulus is the target. However, not all stimulus events provide discriminating information; if either both stimuli flash or neither flashes, the posterior probability remains unchanged. Discriminating information in favor of the correct hypothesis is observed if only the target stimulus flashes but the distractor stimulus does not flash. This occurs with probability *p* = *𝜃*_*T*_(1 − *𝜃*_*D*_). Discriminating information against the correct hypothsesis is observed if only the distractor stimulus flashes but not the target stimulus, which occurs with probability *q* = *𝜃*_*D*_(1 − *𝜃*_*T*_). Hence, task difficulty can be conceptualized as the difference between the probability *p* of observing veridical information and the probability *q* of observing misguiding information. We will use this conceptualization in terms of the probabilities *p* and *q* to investigate the influence of task difficulty on the shape of the RR optimal decision criterion.^1^

**Fig. 2:**
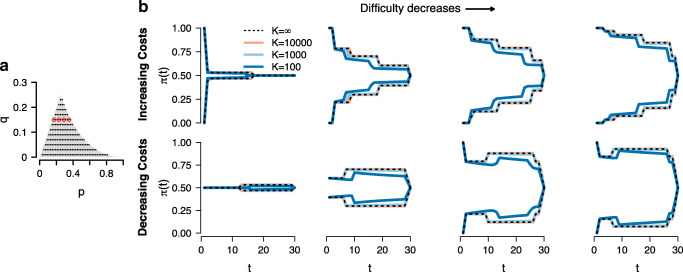
Reward rate optimal decision criterion for different task difficulties and levels of uncertainty. Panel **a** shows the parameter space for our decision environment. The *gray shaded area* indicates the set of possible values of *p* and *q*, *black dots* indicate values of *p* and *q* for which we computed the optimal decision criterion. Panel **b** shows the RR optimal decision criterion for four values of *p* and *q*, indicated by *red circles* in panel **a**. *Dotted black lines* show the optimal decision criterion if the rate parameters *𝜃*_*T*_ and *𝜃*_*D*_ are known exactly ($K=\infty $), *solid lines* show the optimal decision criterion if the rate parameters *𝜃*_*T*_ and *𝜃*_*D*_ have been inferred from *K* = 10000 (*orange*), *K* = 1000 (*light blue*), or *K* = 100 (*dark blue*) prior observations

Panel a of Fig. 2 shows the parameter space for our decision environment. The gray shaded area represents the set of possible values of *p* and *q*. To investigate the influence of task difficulty on the shape of the RR optimal decision criterion, we sampled 201 pairs of values (*p*,*q*) and computed the optimal decision criterion for the reward schemes with increasing and decreasing sampling costs. As the qualitative patterns only depend on the difference between *p* and *q* but not on the specific values of the two parameters, we only discuss the results for a fixed value of *q* and a representative set of four values of *p*, which are indicated by red circles in panel a. We will return to full set of 201 (*p*,*q*) pairs when we compare the expected RR under the optimal decision criterion to the expected RR under a suboptimal static decision criterion below.

Panel b of Fig. 2 shows the RR optimal decision criterion for different task difficulties. The dotted lines show the optimal criterion if the decision-maker knows the rate parameters *𝜃*_*T*_ and *𝜃*_*D*_ exactly. The top row shows the results for the case of increasing sampling costs, with task difficulty decreasing from left to right. As can be seen, in line with the results established in the previous section, increasing sampling costs induce a monotonically decreasing optimal decision criterion, irrespective of the task difficulty. However, the overall height of the decision criterion is lower for higher task difficulties. This result can be understood intuitively as follows. Because discriminating information (i.e., only one of the stimuli flashes) is observed less frequently in a high difficulty task, decision-makers need to acquire more observations to reach a given value of *π*(*t*). At the same time, expected RR decreases as average decision times become longer. Hence, to maintain an acceptable balance between average decision times and expected rewards, decision-makers need to sacrifice some of the expected rewards by accepting a lower value of *π*(*t*) at decision commitment. In the most extreme case, this might result in a premature collapse of the decision criterion to 0.5, as shown in the leftmost panel. In this case of an extremely high task difficulty, at some point during the decision process the decision-maker stands to gain fewer rewards from an additional observation than is incurred in sampling costs by postponing the final decision by one step, so the optimal strategy is to guess.

The bottom row of panel B shows the results for the case of decreasing sampling costs. In line with the results established in the previous section, decreasing sampling costs induce a non-monotonic decision criterion that increases at first but eventually collapses to 0.5 at the time of the decision deadline, irrespective of the task difficulty. Similar to the case of increasing sampling costs, a higher task difficulty results in a lower overall setting of the RR optimal decision criterion. In the case of an extremely high task difficulty, shown in the leftmost panel, the decision criterion might have an initial value of 0.5 so that the optimal strategy is to guess immediately. Due to the high task difficulty, even modest gains in expected rewards require several observations. At the same time, sampling costs are high initially and might therefore outweigh these modest gains in expected rewards.

### Influence of uncertainty about rate parameters

We account for a decision-maker’s uncertainty about the rate parameters by replacing the exact rates *𝜃*_*T*_ and *𝜃*_*D*_ in our sequential sampling model by probability distributions. In particular, we describe the uncertainty about the target and distractor rate by beta distributions with parameters *α*_*T*_ and *β*_*T*_, and *α*_*D*_ and *β*_*D*_, respectively:

5 $$ \theta_{T} \sim B(\alpha_{T}, \beta_{T}), \quad \theta_{D} \sim B(\alpha_{D}, \beta_{D}). $$

The beta distribution is the natural choice for expressing uncertainty about a binomial rate. If *α*_*i*_ = *β*_*i*_ = 1, *i* ∈{*T*,*D*}, the beta distribution is uniform over [0,1], which means that all values for the rates are equally likely. If the distribution parameters are set to positive integer values, the resulting distribution is the posterior distribution a decision-maker obtains from a uniform prior distribution after a total of *α*_*i*_ + *β*_*i*_ observations, of which *α*_*i*_ were positive events (i.e., stimulus *i* flashed *α*_*i*_ times) and *β*_*i*_ were negative events (i.e., stimulus *i* did not flash during the remaining *β*_*i*_ observations). We will model different levels of uncertainty about the rate parameters of the experimental task by setting *α*_*i*_ = *𝜃*_*i*_*K* and *β*_*i*_ = (1 − *𝜃*_*i*_)*K*, where *K* is a positive integer.^2^ The resulting distribution has its mode at the true rate and has its mass more concentrated around the mode for larger values of *K*; this distribution can be interpreted as the posterior distribution obtained from *K* observations of stimulus *i*. We will symbolically write *K* = *∞* for the case where the rate parameters are known exactly.

Due to uncertainty about the rate parameters, the likelihood of any particular type of observation for the target and the distractor stimulus depends on the plausibility of different values of *𝜃*_*T*_ and *𝜃*_*D*_. The decision-maker can account for this uncertainty by marginalizing over all possible values for the rate parameters. The updating rule for the decision-maker’s belief about $\mathcal {H}_{l}$, the probability of the left stimulus being the target, now is:

6 $$ \pi(t) = \frac{\pi(t - 1){{\int}_{0}^{1}}{{\int}_{0}^{1}} f_{\alpha_{T},\beta_{T}}(u)f_{\alpha_{D},\beta_{D}}(v)\lambda_{l}(x_{t},u,v)\mathrm{d}u\mathrm{d}v} {\left( \begin{array}{l} \!\pi(t - 1){{\int}_{0}^{1}}{{\int}_{0}^{1}} f_{\alpha_{T},\beta_{T}}(u)f_{\alpha_{D},\beta_{D}}(v)\lambda_{l}(x_{t},u,v)\mathrm{d}u\mathrm{d}v\\ ~~ +(1 - \pi(t - 1)){{\int}_{0}^{1}}{{\int}_{0}^{1}} f_{\alpha_{T},\beta_{T}}(u)f_{\alpha_{D},\beta_{D}}(v)\lambda_{r}(x_{t}, u, v)\mathrm{d}u\mathrm{d}v \end{array} \right)}. $$

Here $f_{\alpha _{j},\beta _{j}}$, *j* ∈{*T*,*D*}, denotes the probability density function of the beta distribution, and *λ*_*i*_(*x*_*t*_,*u*,*v*) is the likelihood of *x*_*t*_ under $\mathcal {H}_{i}$ given that *𝜃*_*T*_ = *u* and *𝜃*_*D*_ = *v*. A consequence of the marginalization over *𝜃*_*T*_ and *𝜃*_*D*_ is that observations that do not directly discriminate between the target and distractor stimulus nevertheless change the decision-maker’s posterior belief *π*(*t*). If a positive event is observed for both stimuli (i.e., both stimuli flash), for instance, the decision-maker updates the beliefs about the two rate parameters, shifting the mass of the two beta distributions to higher values. This, in turn, may give higher or lower a posteriori plausibility to $\mathcal {H}_{l}$, depending on the decision-maker’s prior beliefs about *𝜃*_*T*_ and *𝜃*_*D*_.

We investigated the influence of uncertainty about the rate parameters on the RR optimal decision criterion for the 201 (*p*,*q*) pairs shown in panel a of Fig. 2. Panel b shows the comparison of the RR optimal decision criterion for different levels of uncertainty for a fixed values of *q* and four representative values of *p*. Dotted lines show the optimal decision criterion when *K* = *∞*, that is, when the rate parameters are known exactly, and solid lines show the optimal criterion for different levels of uncertainty, orange for *K* = 10000, light blue for *K* = 1000, and dark blue for *K* = 100. As can be seen, for lower levels of uncertainty the optimal criterion quickly approaches the criterion when rates are known exactly, and for *K* = 1000 and *K* = 10000 the optimal criterion is visually indistinguishable from the optimal criterion when rates are known exactly. Moreover, the qualitative patterns, even under high uncertainty (i.e., *K* = 100), match the qualitative patterns described in the preceding section for the case where rates are known exactly. We will illustrate the effect uncertainty has on the shape of the RR optimal decision criterion in more detail for the case *𝜃*_*T*_ = 0.38 and *𝜃*_*D*_ = 0.24, and discuss how the RR optimal dynamic decision criterion compares to the best static decision criterion.

**Fig. 3:**
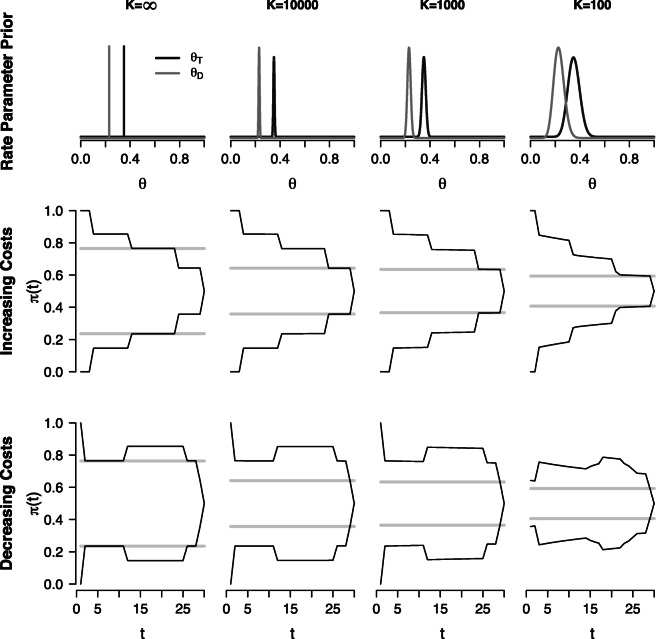
Reward rate optimal decision criterion for different levels of uncertainty. The *top row* shows the prior distributions *𝜃*_*T*_ and *𝜃*_*D*_ for different levels of uncertainty. The *middle* and *bottom row* show the RR optimal dynamic decision criterion (*black solid lines*) and the best static decision criterion (*solid gray lines*) for the case of increasing sampling costs (*middle row*) and for the case of decreasing sampling costs (*bottom row*)

Figure 3 shows how the RR optimal decision criterion changes as uncertainty about the rate parameters increases. The top row of plots shows the prior distributions on *𝜃*_*T*_ and *𝜃*_*D*_ for different values of *K*. For *K* = *∞* the prior distributions are point masses at the true values of the rate parameters. As *K* decreases from left to right, the overlap between the prior distributions for the two rate parameters increases, which means that the two hypotheses, $\mathcal {H}_{l}$ and $\mathcal {H}_{r}$, assign similar likelihood to different types of observations, and are thus harder to discriminate.

The middle row of plots shows the optimal dynamic and static decision criteria for the case of increasing sampling costs. The RR optimal dynamic decision criterion, shown as solid black lines, has the same shape for different levels of uncertainty but collapses more quickly as uncertainty increases from left to right. This quicker collapse is due to the increasing overlap between the prior distributions for *𝜃*_*T*_ and *𝜃*_*D*_ with increasing uncertainty, which causes discriminating information between the two hypotheses to accumulate more slowly. To compensate for this increase in expected decision time and accompanying higher cumulative sampling costs, the decision-maker needs to accept a higher probability of an incorrect decision. The best static decision criterion, shown as solid gray lines, is also set to lower values as uncertainty increases from left to right. Compared to the optimal dynamic decision criterion, the best static decision criterion is set to considerably lower values for the largest part of the decision process but has the same height as the optimal dynamic criterion close to the decision deadline. This might suggest that the best static criterion should result in a larger number of incorrect decisions than the optimal dynamic criterion, and might therefore yield a lower expected reward rate. We will revisit this prediction in the next section.

Finally, the bottom row of plots in Fig. 3 shows the case of decreasing sampling costs. The RR optimal dynamic decision criterion, shown as solid black lines, has a qualitatively similar shape across levels of uncertainty, although it is somewhat less smooth if uncertainty is very high (i.e., *K* = 100). Similar to the results for the case of increasing sampling costs, the optimal dynamic decision criterion is set to overall lower values if uncertainty is high, which is again due to the slower accumulation of discriminating information. The best static decision criterion, shown as solid gray lines, is also set to lower values as uncertainty increases from left to right. Similar to the case of increasing sampling costs, the best static decision criterion is set to overall lower values than the optimal dynamic decision criterion. In the next section we will investigate how the expected RR compares under the optimal dynamic and under the best static decision criterion, and how this relationship depends on sampling costs, task difficulty, and uncertainty about the rate parameters. As the qualitative results in this section were similar for low levels of uncertainty, we will only consider the cases *K* = 1000 and *K* = 100.

To summarize, in the preceding sections we investigated the influence of sampling costs, task difficulty and uncertainty on the shape of the RR optimal decision criterion and on the setting of the best static decision criterion. The results of this analysis show that, first, the main determinant of the shape of the RR optimal decision criterion (i.e., collapsing or expanding) are sampling costs, and, second, task difficulty and uncertainty determine the overall height of the decision criterion but have only a negligible effect on the shape of the decision criterion. Moreover, the best static decision criterion is generally set to a lower value than the optimal dynamic decision criterion, and higher task difficulty and uncertainty result in a lower value of the best static decision criterion.

## Expected reward rate

As the final step of our theoretical analysis we investigated how the factors sampling costs, task difficulty, and uncertainty interact and determine the expected RR. We first computed the RR optimal dynamic decision criterion for the 201 points (*p*,*q*) from the parameter space of our decision environment shown in panel a of Fig. 2. To estimate the expected RR under the optimal dynamic decision criterion for each of the 201 parameterizations of the decision environment, we simulated trials of the experiment and determined the decision time for each simulated trial according to the optimal decision criterion. This procedure continued until we had obtained a minimum of 20,000 trials with an incorrect decision to ensure a good approximation of the decision time distribution. The expected reward rate could then be directly computed as the expected rewards with respect to the decision time distribution. We repeated the same procedure with several settings of a static decision criterion and determined the decision criterion that yielded the highest RR.

**Fig. 4:**
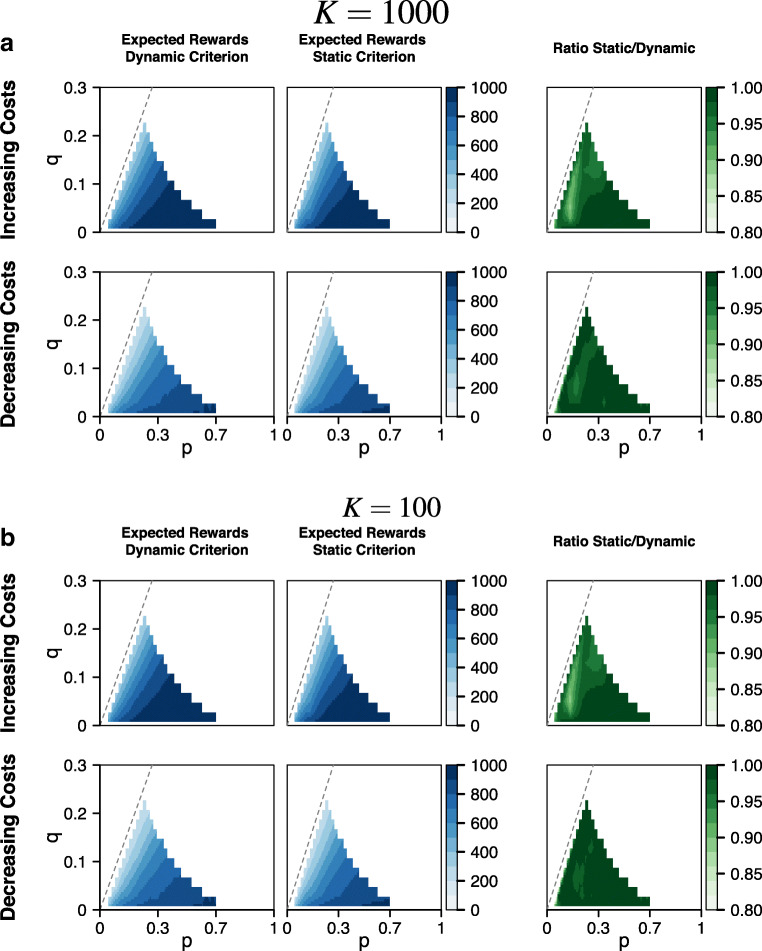
Comparison of expected rewards under static and dynamic decision criteria. Panel **a** shows the comparison for *K* = 1000 for the case of increasing sampling costs (*top row*) and decreasing sampling costs (*bottom row*). Panel **b** shows the comparison for *K* = 100 for the case of increasing sampling costs (*top row*) and decreasing sampling costs (*bottom row*). In each panel, the *left column* shows the expected reward rate for the optimal dynamic decision criterion, the *middle column* shows the expected reward rate for the best static decision criterion, the *right column* shows the ratio of the expected reward rate under the static and dynamic decision criterion. Plots are based on simulated first passage times for a grid of 201 pairs (*p*,*q*) that covers the parameter space of the decision environment

Figure 4 shows how uncertainty, sampling costs, and task difficulty affect the expected RR under the optimal dynamic and under the best static decision criterion. Panel a shows the results for the case where uncertainty about the rate parameters is low (*K* = 1000). The blue heatmaps show the expected RR under the optimal dynamic (left plot) and best static (right plot) decision criterion for increasing (top row) and decreasing sampling costs (bottom row). The dashed line where *p* = *q* in each plot indicates maximum task difficulty. As can be seen, expected RR decreases as task difficulty increases toward the line *p* = *q*. In the case of increasing sampling costs, this decrease in expected RR is quicker under the best static decision criterion than under the optimal dynamic criterion whereas in the case of decreasing sampling costs, the decrease in expected RR appears to be equally fast under both decision criteria. Overall, the expected RR is lower under decreasing sampling costs than under increasing sampling costs.

The green heatmaps show the ratio of the expected RR under the best static decision criterion and the optimal dynamic decision criterion. These heatmaps reveal two important results. First, the loss in expected RR under the static decision criterion relative to the optimal criterion is relatively small. In the case of increasing sampling costs, the expected RR under the static criterion is at least 95% of the maximum RR for 85% of the parameter space of the decision environment, in the case of decreasing sampling costs, the expected RR under the static criterion is at least 95% of the maximum RR for 95% of the parameter space. Our comparison of the shape of the best static and the optimal dynamic decision criterion in the previous section showed that the best static decision criterion was set to considerably lower values for large parts of the decision process and only aligned with the optimal criterion close to the decision deadline. However, this qualitative difference in the shape of the two criteria does not preclude a similar expected RR; if the accumulation of evidence is relatively slow, decision times will tend to lie close to the decision deadline and the shape of the decision criterion early in the decision process will have a negligible influence on the expected RR.

Second, as task difficulty increases, the difference in expected RR between the static and dynamic decision criterion increases. However, for very high task difficulties, the ratio between the expected RR under the static and under the dynamic criterion becomes 1. For extremely high task difficulties the optimal strategy is to guess (i.e., sufficient discriminating information is virtually never observed before the decision deadline), which can be implemented equally well through a dynamic decision criterion that is initially set to 0.5, or a static decision criterion that is set to 0.5 throughout the decision process.

Panel b of Fig. 4 shows the results for the case where uncertainty about the rate parameters is high (*K* = 100). The qualitative patterns are similar to the case of low uncertainty. As can be seen in the blue heatmaps, expected RR decreases as task difficulty increases. In the case of increasing sampling costs, the decrease in expected RR with increasing task difficulty appears to be quicker under the static decision criterion than under the optimal dynamic decision criterion whereas in the case of decreasing sampling costs, the decrease in expected RR seems to be equally fast under both decision criteria. Moreover, expected RR is lower in the case of decreasing sampling costs than in the case of increasing sampling costs. Compared to the results for low uncertainty shown in panel a, higher uncertainty seems to lead to a negligible loss in expected RR.

The ratio of the expected RR under the best static decision criterion and the optimal dynamic decision criterion shown in the green heatmaps shows similar patterns as in the case where uncertainty was low. The loss in expected RR incurred by using the best static instead of the optimal dynamic decision criterion is relatively small. In the case of increasing sampling costs, the best static criterion yields at least 95% of the maximum RR for 85% of the parameter space, and in the case of decreasing sampling costs the best static criterion yields at least 95% of the maximum RR for 95% of the parameter space. Moreover, as task difficulty increases, the expected RR under the static decision criterion decreases relative to the expected RR under the optimal dynamic decision criterion but both decision criteria yield the same RR for extremely high task difficulties. In the case of increasing sampling costs the size of this effect is similar if uncertainty about the rate parameters is low (panel A) or high (panel B). In the case of decreasing sampling costs, however, the change in the ratio of the expected RRs with increasing task difficulty is considerably weaker if uncertainty is high than if uncertainty is low.

## Discussion

In the present work we assessed how sampling costs, task difficulty, and uncertainty about the stochastic structure of the decision environment affect the RR optimality of static and dynamic decision criteria in a typical perceptual decision task. Our analysis showed that the shape of the RR optimal dynamic decision criterion is mainly determined by the sampling costs associated with a delayed final decision. Increasing sampling costs induce a collapsing decision criterion whereas decreasing sampling costs induce an expanding decision criterion, independent of task difficulty and uncertainty about task parameters. Increased task difficulty and uncertainty about task parameters, on the other hand, lead to a lower overall setting of the RR optimal dynamic decision criterion. Our analysis further showed that an a priori suboptimal static decision criterion yielded similar RRs as the optimal dynamic decision criterion across a wide range of task difficulties, under high and low uncertainty, and for increasing as well as decreasing sampling costs. Only task setups with a relatively high difficulty and increasing sampling costs resulted in significant differences between the static and dynamic decision criterion.

An important implication of our theoretical results is that a static decision criterion might be a robust default setting. One of the main motivations for our theoretical analysis was the consistent success of sequential sampling models that assume a static decision criterion by default. Many of the standard experimental paradigms in mathematical psychology create a dynamic decision environment that ought to induce dynamic decision criteria (e.g., Ratcliff and Smith (2004) and Voskuilen et al., (2016)). However, reports of systematic discrepancies between data and models with a static decision criterion are conspicuously absent from the literature. A possible explanation for the success of these models is that a static decision criterion provides a robust default setting that yields near-optimal RRs across a wide range of task setups and levels of uncertainty. At the same time, our results raise the question how representative experimental setups that succeed at inducing a dynamic decision criterion are for the types of decision environments decision-makers encounter in the real world. In our theoretical analysis, decision environments that might reliably induce a collapsing dynamic decision criterion were limited to a narrow range of difficulty levels with increasing sampling costs. Similarly, studies that are regularly cited in support of a default collapsing decision criterion use very specialized experimental setups with strict response deadlines (Miletić and Van Maanen, 2019; Murphy et al., 2016) or long training periods that minimize the decision-maker’s uncertainty about the stochastic properties of the environment, penalty delays for incorrect decisions, and a mixture of task difficulties where the target stimulus is undefined in some trials (i.e., all stimuli are stochastically identical; e.g., Churchland et al., (2008), Hanks et al., (2011), and Drugowitsch et al., (2012)).

Further evidence for the suggestion that a static decision criterion provides a robust default setting is provided by Malhotra et al., (2017). In their analysis, Malhotra et al., considered an expanded judgment task with varying task difficulties but without sampling costs. Using dynamic programming techniques to derive the optimal decision criterion, they found that many mixture proportions of task difficulties used in published experiments yielded nearly constant RR-optimal decision criteria. Only mixtures that included easy and very difficult trials resulted in a markedly collapsing optimal dynamic decision criterion. Moreover, Malhotra et al., (2018) showed that for the particular mixtures of task difficulties used in their experiments, near-optimal RRs could be achieved with a wide range of different slopes of the decision criterion, including a static decision criterion.

A question that is closely related to the default shape of the decision criterion concerns the learning mechanisms through which decision-makers adapt their decision criterion to changing reward structures and stochastic properties of their environment. In the present study we relied on a rudimentary, statistical model that used Bayesian updating to account for decision-makers’ uncertainty about the rate parameters for different stimuli. Moreover, we used computationally intensive dynamic programming techniques to derive the optimal decision criterion. Although this modeling approach has regularly been used in previous studies (e.g., Brown et al., (2009), Drugowitsch et al., (2012), and Malhotra et al., (2018)), its cognitive plausibility is limited. Human decision-makers need to estimate the optimal decision criterion through repeated interactions with their decision environment, which introduces a trade-off between time spent exploiting the current estimate of the optimal decision criterion to obtain rewards, and time spent exploring the environment to refine the decision criterion. Recent experimental studies show that, in a static environment, decision-makers approach RR optimality with practice but their performance remains suboptimal without proper feedback (Evans et al., 2017a, 2017b, 2017c).

Further insight into what degree of RR-optimality human decision-makers can realistically achieve and on what time scale such learning occurs might be gained by incorporating a cognitively plausible learning process into sequential sampling models. Reinforcement learning models (Busemeyer and Stout, 2002; Sutton & Barton, 1998), for instance, have successfully been used to explain the acquisition of optimal decision policies in value-based decision-making (e.g., Ahn et al., (2008), Fridberg et al., (2010), and Steingroever et al., (2014)). Such a combined model, as suggested by Khodadadi et al., (2017), would allow researchers to account for factors such as incomplete exploration, and help quantify the degree of RR-optimality human decision-makers can achieve within a given time frame.

Finally, the theoretical analysis in the present work has focused on a specific experimental paradigm and type of sequential sampling model. Our choice of the experimental paradigm and type of model was based on the types of tasks and models that sparked the recent debate about the RR optimal decision criterion (Cisek et al., 2009; Drugowitsch et al., 2012; Hawkins et al., 2015; Shadlen and Kiani, 2013; Thura et al., 2012; Voskuilen et al., 2016). Here we provided the first systematic evaluation of the theoretical basis for claims that a decreasing dynamic decision criterion should be the default assumption in diffusion-type sequential sampling models (e.g., Shadlen and Kiani (2013)). However, in recent years numerous competitor models have been developed that make different assumptions about the mechanisms that underlie perceptual decision making (e.g., Albantakis and Deco (2009), Bogacz and Gurney (2007), Tsetsos et al., (2012), and Wong and Wang (2006)). Future work should address how claims about the RR optimality of decreasing dynamic decision criteria translate to these models.

## Open practices statement

All programming code for the simulations is available at https://osf.io/cvsrm/. DOI: 10.17605/OSF.IO/CVSRM.

## Footnotes

1 The relationship between the parameterization in terms of *p* and *q*, and in terms of *𝜃*_*T*_ and *𝜃*_*D*_ is not unique. To establish a one-to-one correspondence, we require that *p* < 1/4, in addition to the obvious constraint 0 < *𝜃*_*D*_ < *𝜃*_*T*_ < 1.

2 We round *𝜃*_*i*_ to two decimal places so *α*_*i*_ and *β*_*i*_ are integer-valued for *K* ≥ 100.

## References

[CR1] Ahn W-Y, Busemeyer JR, Wagenmakers E-J, Stout JC (2008). Comparison of decision learning models using the generalization criterion method. Cognitive Science.

[CR2] Albantakis L, Deco G (2009). The encoding of alternatives in multiple-choice decision making. Proceedings of the National Academy of Sciences.

[CR3] Bellman R (2003). Dynamic programming.

[CR4] Boehm U, Hawkins GE, Brown S, Van Rijn H, Wagenmakers E-J (2016). Of monkeys and men: Impatience in perceptual decision-making. Psychonomic Bulletin & Review.

[CR5] Bogacz R, Brown E, Moehlis J, Holmes P, Cohen JD (2006). The physics of optimal decision making: A formal analysis of models of performance in two-alternative forced-choice tasks. Psychological Review.

[CR6] Bogacz R, Gurney K (2007). The basal ganglia and cortex implement optimal decision making between alternative actions. Neural Computation.

[CR7] Brown S, Steyvers M, Wagenmakers EJ (2009). Observing evidence accumulation during multi-alternative decisions. Journal of Mathematical Psychology.

[CR8] Busemeyer JR, Rapoport A (1988). Psychological models of deferred decision making. Journal of Mathematical Psychology.

[CR9] Busemeyer JR, Stout JC (2002). A contribution of cognitive decision models to clinical assessment: Decomposing performance on the Bechara gambling task. Psychological Assessment.

[CR10] Churchland AK, Kiani R, Shadlen MN (2008). Decision-making with multiple alternatives. Nature Neuroscience.

[CR11] Cisek P, Puskas GA, El-Murr S (2009). Decisions in changing conditions: The urgency gating model. The Journal of Neuroscience.

[CR12] DeGroot MH (1969). Optimal statistical decisions.

[CR13] Ditterich J (2006). Stochastic models of decisions about motion direction: Behavior and physiology. Neural Networks.

[CR14] Drugowitsch J, Moreno-Bote R, Churchland AK, Shadlen MN, Pouget A (2012). The cost of accumulating evidence in perceptual decision making. The Journal of Neuroscience.

[CR15] Edwards W (1965). Optimal strategies for seeking information: Models for statistics, choice reaction time, and human information processing. Journal of Mathematical Psychology.

[CR16] Evans, N.J., Bennett, A.J., & Brown, S. D. (2017a). Optimal or not; depends on the task. *Psychonomic Bulletin & Review, 26*(3), 1027–1034. 10.3758/s13423-018-1536-4. 10.3758/s13423-018-1536-4 PMC6557863 30411197

[CR17] Evans, N.J., & Brown, S. D. (2017b). People adopt optimal policies in simple decision-making, after practice and guidance. *Psychonomic Bulletin & Review, 24*(2), 597–606. 10.3758/s13423-016-1135-1. 10.3758/s13423-016-1135-1 27562760

[CR18] Evans, N. J., Hawkins, G. E., Boehm, U., Wagenmakers, E. -j., & Brown, S. D. (2017c). The computations that support simple decision-making: A comparison between the diffusion and urgency-gating models. *Scientific Reports*, *7*, 16433. 10.1038/s41598-017-16694-7. 10.1038/s41598-017-16694-7 PMC5703954 29180789

[CR19] Frazier, P.I., & Yu, A.J. (2008). Sequential hypothesis testing under stochastic deadlines. In J. Platt, D. Koller, Y. Singer, & S. Roweis (Eds.) *Advances in neural information processing systems 20* (pp. 465–472). Cambridge: MIT Press.

[CR20] Fridberg DJ, Queller S, Ahn W-Y, Kim W, Bishara AJ, Busemeyer JR, Stout JC (2010). Cognitive mechanisms underlying risky decision-making in chronic cannabis users. Journal of Mathematical Psychology.

[CR21] Hanks TD, Mazurek ME, Kiani R, Hopp E, Shadlen MN (2011). Elapsed decision time affects the weighting of prior probability in a perceptual decision task. Journal of Neuroscience.

[CR22] Hawkins GE, Forstmann BU, Wagenmakers E-J, Ratcliff R, Brown SD (2015). Revisiting the evidence for collapsing boundaries and urgency signals in perceptual decision making. Journal of Neuroscience.

[CR23] Heath RA (1981). A tandem random-walk model for psychological discrimination. British Journal of Mathematical and Statistical Psychology.

[CR24] Huang, Y., & Rao, R.P.N. (2013). Reward optimization in the primate brain: A probabilistic model of decision making under uncertainty. PLoS One. 10.1371/journal.pone.0053344. 10.1371/journal.pone.0053344 PMC3551910 23349707

[CR25] Irwin FW, Smith WAS, Mayfield JF (1956). Tests of two theories of decision in an “expanded judgment” situation. Journal of Experimental Psychology.

[CR26] Kahneman D, Tversky A (1979). Prospect theory: An analysis of decision under risk. Econometrica.

[CR27] Khodadadi A, Fakhari P, Busemeyer JR (2017). Learning to allocate limited time to decisions with different expected outcomes. Cognitive Psychology.

[CR28] Malhotra G, Leslie DS, Ludwig CJH, Bogacz R (2018). Time-varying decision boundaries: Insights from optimality analysis. Psychonomic Bulletin & Review.

[CR29] Malhotra G, Leslie DS, Ludwig CJH, Bogacz R (2017). Overcoming indecision by changing the decision boundary. Journal of Experimental Psychology: General.

[CR30] Miletić S, Van Maanen L (2019). Caution in decision-making under time pressure is mediated by timing ability. Cognitive Psychology.

[CR31] Moran R (2015). Optimal decision making in heterogeneous and biased environments. Psychonomic Bulletin and Review.

[CR32] Murphy PR, Boonstra E, Nieuwenhuis S (2016). Global gain modulation generates time-dependent urgency during perceptual choice in humans. Nature Communications.

[CR33] Rao, R. P. N. (2010). Decision making under uncertainty: a neural model based on partially observable Markov decision processes. Frontiers in Computational Neuroscience, 4. 10.3389/fncom.2010.00146. 10.3389/fncom.2010.00146 PMC2998859 21152255

[CR34] Rapoport A, Burkheimer GJ (1971). Models for deferred decision making. Journal of Mathematical Psychology.

[CR35] Ratcliff R (1978). A theory of memory retrieval. Psychological Review.

[CR36] Ratcliff R, Smith PL (2004). A comparison of sequential sampling models for two-choice reaction time. Psychological Review.

[CR37] Sanders AF, Ter Linden W (1967). Decision making during paced arrival of probabilistic information. Acta Psychologica.

[CR38] Savage LJ (1954). The foundations of statistics.

[CR39] Shadlen MN, Kiani R (2013). Decision making as a window on cognition. Neuron.

[CR40] Standage, D., You, H., Wang, D. -H., & Dorris, M. C. (2011). Gain modulation by an urgency signal controls the speed-accuracy trade-off in a network model of a cortical decision circuit. Frontiers in Computational Neuroscience, 5. 10.3389/fncom.2011.00007. 10.3389/fncom.2011.00007 PMC3042674 21415911

[CR41] Steingroever H, Wetzels R, Wagenmakers E-J (2014). Absolute performance of reinforcement-learning models for the Iowa Gambling Task. Decision.

[CR42] Stone M (1960). Models for choice-reaction time. Psychometrika.

[CR43] Summerfield, C., & Tsetsos, K. (2012). Building bridges between perceptual and economic decision-making: Neural and computational mechanisms. Frontiers in Neuroscience. 10.3389/fnins.2012.00070. 10.3389/fnins.2012.00070 PMC3359443 22654730

[CR44] Sutton RS, Barton AG (1998). Reinforcement learning: An introduction.

[CR45] Thura D, Beauregard-Racine J, Fradet C-W, Cisek P (2012). Decision making by urgency gating: Theory and experimental support. Journal of Neurophysiology.

[CR46] Tsetsos, K., Gao, J., Mcclelland, J. L., & Usher, M. (2012). Using time-varying evidence to test models of decision dynamics: bounded diffusion vs. the leaky competing accumulator model. Frontiers in Neuroscience, 6. 10.3389/fnins.2012.00079. 10.3389/fnins.2012.00079 PMC3372959 22701399

[CR47] Voskuilen C, Ratcliff R, Smith PL (2016). Comparing fixed and collapsing boundary versions of the diffusion model. Journal of Mathematical Psychology.

[CR48] Wagenmakers E-J, Ratcliff R, Gomez P, McKoon G (2008). A diffusion model account of criterion shifts in the lexical decision task. Journal of Memory and Language.

[CR49] Wald A (1945). Sequential tests of statistical hypotheses. The Annals of Mathematical Statistics.

[CR50] Wald A, Wolfowitz J (1948). Optimum character of the sequential probability ratio test. The Annals of Mathematical Statistics.

[CR51] Wallsten TS (1968). Failure of predictions from subjectively expected utility theory in a Bayesian decision task. Organizational Behavior and Human Performance.

[CR52] Wong K-F, Wang X-J (2006). A recurrent network mechanism of time integration in perceptual decisions. The Journal of Neuroscience.

[CR53] Yap MJ, Sibley DE, Balota DA, Ratcliff R, Rueckl J (2015). Responding to nonwords in the lexical decision task: Insights from the English Lexicon Project. Journal of Experimental Psychology: Learning, Memory, and Cognition.

